# Activating ligands of Uncoupling protein 1 identified by rapid membrane protein thermostability shift analysis

**DOI:** 10.1016/j.molmet.2022.101526

**Published:** 2022-06-09

**Authors:** Riccardo Cavalieri, Marlou Klein Hazebroek, Camila A. Cotrim, Yang Lee, Edmund R.S. Kunji, Martin Jastroch, Susanne Keipert, Paul G. Crichton

**Affiliations:** 1 Biomedical Research Centre, Norwich Medical School, University of East Anglia, Norwich Research Park, Norwich, NR4 7TJ, United Kingdom; 2 Department of Molecular Biosciences, The Wenner-Gren Institute, Stockholm University, SE-106 91, Stockholm, Sweden; 3 Medical Research Council, Mitochondrial Biology Unit, University of Cambridge, Cambridge Biomedical Campus, Hills Road, Keith Peters Building, CB2 0XY, United Kingdom

**Keywords:** Ligand binding, Thermal stability assay, Differential scanning fluorimetry, Brown adipose tissue, Proton transport, Energy expenditure, Mitochondrial carrier, UCP1, Uncoupling protein 1, 12MNG, dodecyl maltose neopentyl glycol (2,2-didecylpropane-1,3-bis-β-d-maltopyranoside), OLPA, oleoyl-l-α-lysophosphatidic acid, TTNPB, 4-[(E)-2-(5,6,7,8-Tetrahydro-5,5,8,8-tetramethyl-2-naphthalenyl)-1-propenyl]benzoic acid, C7, heptanoic acid, C12, dodecanoic acid, C19, nonadecanoic acid, C6-S, hexane sulfonate, C11-S, undecane sulfonate, C18-S, octadecane sulfonate

## Abstract

**Objective:**

Uncoupling protein 1 (UCP1) catalyses mitochondrial proton leak in brown adipose tissue to facilitate nutrient oxidation for heat production, and may combat metabolic disease if activated in humans. During the adrenergic stimulation of brown adipocytes, free fatty acids generated from lipolysis activate UCP1 via an unclear interaction. Here, we set out to characterise activator binding to purified UCP1 to clarify the activation process, discern novel activators and the potential to target UCP1.

**Methods:**

We assessed ligand binding to purified UCP1 by protein thermostability shift analysis, which unlike many conventional approaches can inform on the binding of hydrophobic ligands to membrane proteins. A detailed activator interaction analysis and screening approach was carried out, supported by investigations of UCP1 activity in liposomes, isolated brown fat mitochondria and UCP1 expression-controlled cell lines.

**Results:**

We reveal that fatty acids and other activators influence UCP1 through a specific destabilising interaction, behaving as transport substrates that shift the protein to a less stable conformation of a transport cycle. Through the detection of specific stability shifts in screens, we identify novel activators, including the over-the-counter drug ibuprofen, where ligand analysis indicates that UCP1 has a relatively wide structural specificity for interacting molecules. Ibuprofen successfully induced UCP1 activity in liposomes, isolated brown fat mitochondria and UCP1-expressing HEK293 cells but not in cultured brown adipocytes, suggesting drug delivery differs in each cell type.

**Conclusions:**

These findings clarify the nature of the activator-UCP1 interaction and demonstrate that the targeting of UCP1 in cells by approved drugs is in principle achievable as a therapeutic avenue, but requires variants with more effective delivery in brown adipocytes.

## Highlights

• UCP1 thermostability shifts can identify fatty acid activator binding.
• Activators destabilise UCP1, binding as transport substrates in the mechanism.
• UCP1 has a wide ligand specificity for activators.
• Screens identify new activators, including the licenced drug ibuprofen.
• Drug-targeting UCP1 in cells is viable to increase energy expenditure.

## 1 Introduction

Brown adipose tissue of mammals has the specialised ability to oxidise nutrients to generate heat for defence of body temperature against the cold [1], and may help combat metabolic disease in humans. Brown fat occurrence in adults correlates with leanness [[2], [3], [4]] where activation of the tissue increases energy expenditure, lipid turnover, glucose disposal and insulin sensitivity, consistent with a positive impact on systemic metabolism and health [[5], [6], [7], [8]]. Thermogenesis by brown fat is achieved through the activation of Uncoupling protein 1 (UCP1), a 33 kDa mitochondrial carrier that catalyses the leak of protons across the mitochondrial inner membrane, dissipating the protonmotive force to release energy as heat at the expense of ATP synthesis (see [9] for review). Following the adrenergic stimulation of brown adipocytes, e.g. by norepinephrine following cold exposure, intracellular cAMP-dependent signalling initiates lipolysis and the release of long chain fatty acids, which directly interact to activate UCP1, overcoming the inhibition of the protein by cytosolic purine nucleotides [10]. Methods to encourage brown fat development and the activation of UCP1 in the absence of physiological stimuli is a therapeutic strategy to combat diabetes, obesity and related diseases, e.g., by pharmacological activation of adipocyte β_3_-adrenergic receptors [6,11].

How fatty acids induce proton leak by UCP1 is debated (see [9] for biochemical models). Fatty acid anions may act as a co-factor to complete a proton transport channel within the protein [12] or, instead, act as a transport substrate that is exported by UCP1 to flip back directly across the mitochondrial inner membrane in a protonated form, independent of the protein, to give a net proton transfer [13,14]. Alternatively, fatty acids in both protonated and deprotonated forms may be transported by UCP1, where long chain species remain bound to the protein following transport across the membrane, allowing just protons to be chaperoned down their electrochemical gradient [15]. The fatty acids may also compete directly or act allosterically to remove inhibitory purine nucleotides, which bind to UCP1 from the cytosolic side [16].

UCP1 is a member of the mitochondrial carrier family of metabolite transporters, which all share the same basic fold and membrane topology. They are comprised of three ∼100 amino acid repeat domains, each composed of two transmembrane α-helices linked by a matrix loop and small α-helix, which together form a six transmembrane helix barrel arrangement with three-fold pseudosymmetry [17,18]. Our purification studies of native UCP1 have revealed that the protein is a monomer and tightly binds three cardiolipin molecules to maintain stability, similar to other mitochondrial carriers [19]. Though to what degree UCP1 employs a common carrier metabolite transport mechanism, as recently resolved for the ADP/ATP carrier [20], for proton leak is not clear. The protein has been postulated to employ a mechanistically distinct process for proton leak [21]. UCP1 has been studied for many years, yet the location and molecular nature of the fatty acid binding site has not been clarified. For membrane proteins in general, there has been a lack of efficient methods to report on ligand interactions due to the practical complications imposed by membrane hydrophobicity and common need for detergents. Fatty acids add further challenges as ionic surfactants, binding non-specifically with potential to denature proteins. Information on fatty acid activator binding to UCP1 has largely been indirect and inferred from changes in nucleotide binding or activity measurements (e.g. [16,22,23]). An improved understanding of the activator interaction may provide therapeutic avenues to target UCP1 directly for activation to engage thermogenesis.

Here, we demonstrate the efficient detection of activator interactions with purified UCP1 through protein thermal stability shift analysis. The approach not only informs on the nature of the interaction process but provides an effective screening avenue to identify novel molecules with potential to activate UCP1 in cells.

## 2 Materials and methods

### 2.1 Materials

Dodecyl maltose neopentyl glycol (12MNG) detergent was obtained from Generon (Slough, UK). Lipids were obtained from Avanti Polar Lipids (AL, USA). Compounds were obtained from Cambridge Bioscience (Cambridge, UK) or Sigma–Aldrich (Merck Group). Unless otherwise stated, all other chemicals were obtained from Sigma–Aldrich (Merck Group) or Thermo Fisher Scientific.

### 2.2 Tissue collection and isolation of mitochondria

Perirenal brown fat was collected from newborn lambs (10–40 g per animal) that had died of natural causes at local Norfolk and Suffolk farms, and flash frozen in liquid nitrogen for storage until further use. Brown adipose tissue mitochondria were isolated following previously described methods [19], adapted from Ref. [24]. Final mitochondrial samples (30–40 mg/mL protein) were flash frozen and stored in liquid nitrogen until further used.

Brown fat of WT and UCP1 KO mice ([25]; age 8–10 weeks) was collected and put in ice cold buffer (250 mM Sucrose, 10 mM TES, 2 mM EDTA and 1% defatted BSA (w/v), pH 7.2). The tissue was minced in buffer, on ice, with fine scissors and homogenised using a glass-Teflon homogeniser. The homogenate was filtered through nylon mesh and centrifuged (10 min, 8740 *g*, 4 °C). Supernatant was discarded and the lipid remaining on the inside walls of the tube was removed using tissue paper. The pellet was resuspended in buffer (250 mM Sucrose, 10 mM TES, 1 mM EGTA, 0.8% defatted BSA (w/v), pH 7.2) and centrifuged (10 min, 950 *g*, 4 °C). The resulting supernatant was collected in a new tube and centrifuged (10 min, 8740 *g*, 4 °C). The supernatant was discarded, and the pellet was resuspended in isolation buffer (100 mM KCl, 20 mM TES, 1 mM EGTA, pH 7.2). The solution was centrifuged (10 min, 8740 *g*, 4 °C), and the final mitochondrial pellet was resuspended in a minimum volume of buffer (100 mM KCl, 20 mM TES, 1 mM EGTA, pH 7.2). Mitochondrial protein content was determined using the Biuret method.

### 2.3 Gene sequencing

The coding regions of the ovine UCP1 gene were verified by amplification of UCP1 exons from genomic DNA by polymerase chain reaction (PCR) and DNA sequencing.

Genomic DNA was isolated from ovine brown adipose tissue by phenol–chloroform extraction. ∼300 mg of frozen tissue was ground, thawed and homogenised in 1.0 mL buffer (50 mM Tris–HCl pH 8, 25 mM EDTA, 400 mM NaCl, 1% SDS, 0.2 μg/μL proteinase K) and incubated at 65 °C for 2 h. The sample was centrifuged (5 min, ∼16 000 *g*) and the supernatant recovered avoiding the pellet and top fat layer. 1/10 volumes of 5 M K-acetate (pH 7.5) was added and the sample incubated on ice for 30 min, followed by centrifugation (10 min, ∼16 000 *g*). The supernatant was recovered in a fresh tube and vigorously mixed with an equal volume of phenol: chloroform: isoamyl alcohol (25:24:1). Following centrifugation (5 min, ∼16 000 *g*), the top layer was recovered (using a cut-off pipette tip) and a volume of chloroform added and vigorously mixed to the remaining bottom layer. Following re-centrifugation, the resulting top layer was recovered and pooled with the first top layer taken. Two volumes of ice-cold ethanol were mixed in and the sample incubated at −20 °C for 30 min to precipitate the DNA. Following centrifugation (10 min, 16 000 *g*), a DNA pellet was recovered and washed in 75% ethanol, re-centrifuged followed by a repeated wash. The final pellet was dried under a stream of nitrogen gas to remove excess water/ethanol and resuspended in nuclease-free water before quantification and further use.

Ovine genome sequence information ([26], gi957807226: 16832912-16839562, via NCBI: https://www.ncbi.nlm.nih.gov/gene/) was used to generate the primer pairs (below) to amplify the 6 exons of the ovine UCP1 gene by PCR. PCR products were verified and purified by standard agarose gel electrophoresis methods utilising DNA sample clean up kits (Qiagen), and were sequenced using the same primers through commercial services (Source Bioscience, UK). The resulting data allowed the coding sequence for lamb UCP1 to be compiled based on established splicing (see Supp. Fig. 1), which highlighted a TAT codon (rather than TGT) encoding tyrosine at position 55 in the protein.

**Exon 1**
F1 AAGTCGGAGAGGACGGGTCT
R1 TGGTAAAACCGAAGCCAGAGT
**Exon 2**
F2 AGATGTTGCTGAGAAGGCGAA
R2 CCTGCAAGCTCTCCTGTGAT
**Exon 3 and 4**
F3 TGCACCAGCATTAGGGAAGT
R3 ATACAGCTGCATCTTAGTTTTGTT
**Exon 5**
F4 GTCATGCCCTAAGGACAGAGG
R4 TGCACATTCTAAGGCTGCTCA
**Exon 6**
F5 TCCTATACTCCATTCAGAAAGCAA
R5 TGAATGTTTTGCTTTCCCTTCCT

### 2.4 Purification of native UCP1

UCP1 was purified using hydroxyapatite and thiopropyl sepharose chromatography media based on methods described in Refs. [19,27]. 100 mg of thawed mitochondrial membranes were centrifuged (110 000 *g* for 20 min, °C) and the pellet resuspended and solubilised in 3% (v/v) Triton X-100 in purification medium (20 mM MOPS-NaOH pH 6.7, 20 mM Na_2_SO_4_, 0.16 mM EDTA) at 4 °C for 20 min with gentle mixing. Insoluble material was pelleted by repeated ultracentrifugation and the supernatant recovered and loaded onto an hydroxyapatite column (7 g dry-weight resin, Biorad 130-0420, hydrated and packed in a disposable chromatography column, Biorad 732-1010, and pre-equilibrated in purification medium). Following column loading (0.7 mL/min), the flow was paused and the column incubated for 15 min at room temperature. Following addition of purification medium and re-starting the flow, ∼11 mL of sample containing UCP1 was collected after the first 10 mL of eluate. The protein was immobilised and purified further by covalent chromatography. The sample was supplemented with 50 mM Tris pH 8.0 and 1 mM EDTA and applied to ∼300 mg of thiopropyl Sepharose 6B (Sigma T8387; pre-hydrated in degassed water) in an empty PD-10 column, and rotated for 1 h at 4 °C to allow UCP1 to bind to the resin. After settling, the column containing the sample was attached to a peristaltic pump and the resin carefully washed (∼2.5 mL/min flow) with 30 mL wash buffer 1 (20 mM Tris pH 8, 0.5% 12MNG, ±0.5 mg/mL tetraoleoyl cardiolipin, 50 mM NaCl, 1 mM EDTA), followed by 30 mL wash buffer 2 (20 mM Tris pH 8, 0.005% 12MNG, ±0.005 mg/mL tetraoleoyl cardiolipin, 50 mM NaCl, 1 mM EDTA) to facilitate contaminant removal and detergent exchange. The column was detached from the pump, briefly centrifuged (500 *g*, 1 min) to remove excess liquid, and 1 mL of elution buffer (wash buffer 2 supplemented with 150 mM dithiothreitol) added to the resin before agitation in a cold room for 15 min. UCP1 was eluted by centrifugation (500 *g*, 1 min) and a further 0.8 mL of elution buffer added to the resin followed by repeated incubation. Following elution by centrifugation (2000 *g*, 2 min), samples were pooled and the protein exchanged into sample buffer (20 mM Tris pH 8, 0.005% 12MNG, ±0.005 mg/mL tetraoleoyl cardiolipin) using a PD-10 column (17-0851-01; GE Healthcare) and protein quantified (BCA assay, Thermo Scientific) before flash freezing and storage in liquid nitrogen. Note, the supplementation of buffers with cardiolipin lipid, where indicated, was only carried out for UCP1 preparations used for liposome reconstitution experiments.

### 2.5 Protein thermostability shift measurements

Protein thermostability measurements were made using a fluorescence based assay suitable for membrane proteins [28] developed for enhanced and rapid use on a rotary qPCR machine with ‘melt’ analysis software [29]. In the assay, CPM dye (7-Diethylamino-3-(4′-Maleimidylphenyl)-4-Methylcoumarin; Invitrogen D346) reacts with protein thiols as they become solvent exposed due to thermal denaturation to give a fluorescent adduct, which is used to monitor protein unfolding during an applied temperature ramp. In preparation for use, CPM dye stock (5 mg/mL; stored in DMSO at −80 °C) was thawed and diluted 50-fold into assay buffer (20 mM Hepes pH 7.5, 0.1% 12MNG) and incubated in the dark for 10–15 min at room temperature. Per test, 2 μg of UCP1 protein was diluted into assay buffer, with test compounds added where required, to 45 μL in 200 μL thin-walled PCR tubes. Unless stated otherwise, 1 μL of test compound was added from a 50× stock or the appropriate solvent control (DMSO, ethanol or water). 5 μL of the diluted CPM solution was mixed into each test sample, which was subsequently incubated for 10–15 min on ice. Samples were placed in a Rotor-Gene Q HRM 2-plex PCR cycler (36 position rotor) and subjected to a high resolution melt procedure (‘HRM’ channel: *λ*_ex_ 440–480 nm, *λ*_em_ 505–515 nm), with the instrument software set to give a required pre-measurement holding step (90 s, beginning at 18 °C) followed by a temperature increase from 25 to 90 °C in 1 °C increments (‘wait between reading’ set to 4 s), corresponding to a temperature ramp of 5.6 °C/min. Unless stated otherwise, protein unfolding profiles were analysed using the instrument software, where the peak in the derivative provided a ‘melt temperature’ (*T*_m_) as a measure of relative protein stability. Typically, up to 18 tests were carried out per run, where *T*_m_ values gained in the presence of a given test compound/condition were subtracted from the corresponding ‘no compound’ solvent control to provide thermostability shift values (Δ*T*_m_) associated with compound–protein interaction.

### 2.6 Liposome reconstitution and proton flux measurements

UCP1 was reconstituted into liposomes based on methods described previously [19,30], with adaptions for the use of the probe SPQ (6-Methoxy-N-(3-Sulfopropyl)Quinolinium, Invitrogen, M440) for proton transport measurements [13,31]. Ten milligrams of lipid (18:1 phosphatidylcholine supplemented with 5% 18:1 cardiolipin) was dried from storage in chloroform under a nitrogen stream, resolubilised in methanol, and re-dried to a smear in a 1.5-mL Eppendorf tube. The lipids were mixed with water and reagent stocks to form an emulsion and solubilised with 55 μL 25% (vol/vol) C_10_E_5_ detergent (Sigma 76436) on ice before addition of 20 μg of purified UCP1, to give a final ‘internal medium’ buffer composition of 100 mM K^+^ (phosphate salt, pH 7.5), 0.1 mM tetraethyl ammonium (TEA^+^; phosphate salt, pH 7.5), 30 mM TES (TEA^+^ salt, pH 7.5), 0.5 mM EDTA (TEA^+^ salt, pH 7.5) and 2 mM SPQ, with a final volume equivalent to 0.6 mL in the absence detergent. The detergent was removed through addition of 4 × 30 mg and a further 4 × 60 mg of adsorbent beads (Bio-Beads SM-2) in 20 min intervals with gentle mixing in a cold room. The resulting proteoliposomes were separated from the biobeads by using empty spin columns (Bio-Rad 732–6204) and treated with 40 mM methyl-β-cyclodextrin (Sigma C4555; 5–10 min on ice) to sequester fatty acids, and exchanged into an external buffer [0.1 mM K^+^ (phosphate salt, pH 7.5), 100 mM TEA^+^ (phosphate salt, pH 7.5), 30 mM TES (TEA^+^ salt, pH 7.5), 0.5 mM EDTA (TEA^+^ salt, pH 7.5)] using a PD10 column. Following column loading (0.6 mL followed by 1.9 mL of buffer), the liposomes were collected in the first 1.4 mL of elution and stored on ice until further use.

Proton uptake into liposomes was tracked through changes in fluorescence of liposome-entrapped SPQ that is quenched specifically by the anionic component of the buffer (e.g. TES^−^), which changes in concentration in response to proton movement (see [31]). Measurements were made in a Cary Eclipse spectrofluorometer (*λ*_ex_ 334 nm, *λ*_em_ 443 nm) at 25 °C, where 75 μL of proteoliposomes were diluted to 500 μL in external buffer (see above) in a quartz cuvette, with 100 μM test compound, or 1 mM GDP, added from 100-fold or greater stocks solutions (i.e. in ≤5 μL volume), unless stated otherwise. Compound stocks were made up in water or ethanol (DMSO could not be used with the assay), where control traces included the equivalent volume of solvent alone. For each measurement, a signal was recorded for 40 s before a membrane potential was induced through the addition of the K^+^ ionophore valinomycin (2.5 μM) to drive proton uptake (added from a 250× stock in ethanol via a disposable inoculation loop). After a further 60 s, 1 μM CCCP was introduced in a similar manner to reveal the maximum proton uptake capacity of the system. Calibration of changes in signal to changes in internal proton concentration was achieved for each proteoliposome batch by diluting a 75 μL aliquot into internal buffer assay medium (rather than external buffer) and recording the signal changes associated with multiple 1 μL additions of 1 M H_2_SO_4_ in the presence 10 μM nigericin. The observed linear trends in Stern–Volmer plots (1/F vs δ[H^+^]) were modelled by linear regression to allow signal conversion in the corresponding progress curves as described in Ref. [31]. The initial rate of change of proton concentration associated with UCP1 activity was estimated by fitting data 10 s before to 59 s after the addition of valinomycin to the exponential function ‘plateau and one phase association’ in GraphPad Prism software. The liposome internal volume was calculated by diluting a 75 μL aliquot into buffer with 0.2% Triton X-100 detergent to release entrapped SPQ, which was quantified by fluorescence using a standard curve after further SPQ additions of known concentration. Progress curves were corrected for internal volume to give the total number of protons moved per sample aliquot, per unit time for UCP1 rate estimations.

### 2.7 Ligand-based modelling perspective

Compounds were computationally aligned and analysed through ligand-based pharmacophore modelling software (Ligandscout). Once uploaded, the relevant compound structures were set to ionised acids and energy minimised. The set was clustered and conformers generated for each (‘maximum number of conformers’ set to 1000; ‘timeout’ to 1200 s, other settings to default). Pharmacophore models were subsequently generated, overnight where necessary, using the best fit and atom overlap with merged feature pharmacophore options to discern common features. For UCP1 proton leak activators, the following ten compounds were used: 4-heptylbenzoic acid; bromododecanoic acid; dodecanoic acid; ibuprofen; nonadecanoic acid; oleic acid; retinoic acid; tetradecylthioacetic acid; TTNPB; TUG-891. For the wider group of destabilising ligands of UCP1, the following additional 11 compounds were also included: 1-octadecanesulfonic acid; 4-heptylbenzoic acid; 6-phenyl-hexanoic acid; acitretin; adapalene; agaric acid; celecoxib; medica-16; perfluorotridecanoic acid; tazarotene; tetradecadioic acid; undecane-1-sulfonate. In each case, the top outcome models were selected (scoring 0.81 and 0.77 for the activator and wider destabilising ligand pharmacophore, respectively). Molecules were aligned by features; representative conformers are shown.

### 2.8 Cell culture methods and immortalisation of mouse brown adipocytes

UCP1-expressing HEK293 cells were generated as described previously [32], and cultured in growth medium consisting of DMEM, high glucose (Gibco) with the addition of 10% Fetal Bovine Serum (Gibco), 1% Penicillin-Streptomycin solution (10 000 U/mL, Gibco) and 1% Geniticin selective Antibiotic (G418 Sulfate, 50 mg/mL, Gibco). Cells were trypsinized from culture dishes and seeded in appropriate amounts for experiments.

The isolation of the stromal vascular (SV) fraction from the interscapular brown adipose tissue (BAT) pad of WT and UCP1 KO mice (6–8 weeks old) was performed as follows. BAT pads were minced and then digested for 30 min at 37 °C (DMEM/F12 plus glutamax, 0.15% (w/v) collagenase Type IV, 1% BSA). The homogenate was filtered through a 100 μm filter and rinsed with DMEM/F12. Thereafter, the cells were centrifuged at 400 *g* for 10 min. The pellet was washed once more and centrifuged at 400 *g* for 10 min. The cells were re-suspended in growth media (DMEM/F12 plus glutamax, 10% FCS, 1% penicillin/streptomycin), plated and cultured. After 24 h, pre-adipocytes were immortalised using the SV40 large T-antigen. 48 h later, the cells were split, seeded into new flasks, allowed to grow to 60%–70% confluence and either further split or frozen into aliquots using freezing media (Growth media, 10% DMSO).

### 2.9 Extracellular flux measurements

HEK293 cells were seeded onto XF96 cell culture microplates (15k cells per well). The next day, the medium was changed to XF Assay medium (Agilent, 102353-100) with the addition of 10 mM Glucose, 10 mM Pyruvate and 0.4% defatted BSA (w/v) at pH 7.5 and incubated for an hour in an air incubator without CO_2_ at 37 °C prior to the experiment. Three assay cycles (1 min mix, 2 min wait, 3 min measuring period) were used to determine basal respiration, followed by three assay cycles after oligomycin injection (4 μg/mL) to determine proton leak respiration. For the measurement of UCP1 activity, TTNPB (15 μM), ibuprofen (0.05, 0.25 and 0.5 mM) or a buffer control were used, followed by injection of chemical uncoupler (dinitrophenol, DNP at 100 μM) to determine maximal respiration and a final injection of rotenone (5 μM) and antimycin A (2 μM) to determine non-mitochondrial respiration. The measurements were performed in multiple wells on four independent experimental days (n = 17–23 wells per group).

For brown adipocytes, immortalised preadipocytes were plated onto XF96 cell culture micro plates (12K per well) and allowed to grow to 90%–100% confluence. At confluence, cell differentiation was started using differentiation cocktail tailored to brown adipocytes (Growth media, 5 μM dexamethasone, 0.5 mM IBMX, 1 μM rosiglitazone, 0.5 μg/mL insulin, 125 μM indomethacin, 1 nM T3) for 2 days, followed by a change to continuation medium (Growth media, 1 μM rosiglitazone, 0.5 μg/mL insulin, 1 nM T3) for 2 days, and a change to differentiation medium (Growth media, 0.5 μg/mL Insulin, 1 nM T3) for 2 days. On day six of differentiation the cells were used for cellular respiration assays. Adipocytes were switched to XF Assay medium (Agilent, 102353-100) with the addition of 20 mM glucose, 2 mM glutamine and 0.4% defatted BSA (w/v) at pH 7.2. The cells were incubated for 10 min in an air incubator without CO_2_ at 37 °C. Four assay cycles (2 min mix, 0 min waiting and 2 min measuring period) were used to determine basal respiration, followed by three assay cycles after oligomycin injection (5 μg/mL) to determine proton leak respiration. For the measurement of UCP1 activation, isoproterenol (0.5 μM), ibuprofen (0.05, 0.25 and 0.5 mM) or a buffer control were used, followed by dinitrophenol (DNP; 150 μM) injection to determine maximal respiration and a final injection of rotenone (5 μM) and antimycin A (2 μM) to determine non-mitochondrial respiration. These measurements were performed in multiple wells on three independent experimental days (n = 9 wells per group).

### 2.10 Mitochondrial membrane potential measurements

Measurements of Safranin O fluorescence were done in a 96-well plate with the Clariostar (*λ*_ex_ 533-15 nm, *λ*_em_ 576-20 nm, bottom optic, gain 1670, focal height of 5.4 mm, cycle time of 18 s and orbital shaking (30 s at 300 rpm) before the first measurement). Per well, 40 μg of mitochondria were used along with 200 μL of pre-warmed KHE buffer (50 mM KCl, 5 mM TES, 2 mM MgCl_2_, 4 mM KH_2_PO_4_, 1 mM EGTA, 0.8% defatted BSA (w/v), pH 7.2) mixed with safranin O (10 μM), nigericin (100 nM), oligomycin (1 μg/mL) and rotenone (4 μM). A baseline measurement was taken for 10 cycles. After, succinate (5 mM) was added to each well, and the measurement continued for 15 cycles. For the measurement of UCP1 activity, palmitate (100 μM) and ibuprofen (250 μM) were used, followed by injection of GDP (1 mM). Lastly, FCCP was titrated in all wells until maximum fluorescence was reached (steps of 6 μM, 3 μM and 3 μM), with 10 measuring cycles after each addition. The measurements were performed in multiple wells on two independent experimental days (n = 6 wells per group).

### 2.11 Western blotting

During cultivation, WT and UCP1 KO immortalised brown adipocytes were collected for protein extraction using RIPA-based lysis buffer (150 mM NaCl, 1% IGEPAL CA-630, 0.5% sodium deoxycholate, 0.1% SDS, 50 mM Tris, pH 8.0) with a phosphatase/protease inhibitor. Differentiated adipocytes (day 6) were scraped off the 6-well plate and incubated on ice for 30 °C. Thereafter, samples were centrifuged for 30 min at 18.000 *g* at 4 °C, and the supernatant was collected. Protein concentration was quantified with a Bradford assay (Sigma). The following primary antibodies were used: UCP1 (1:2000, Abcam, ab23841) and α Tubulin Antibody (1:2000, Santa Cruz, sc-23948). The following Horseradish-peroxidase-conjugated secondary antibodies were used: Goat Anti-Rabbit IgG H&L (HRP) (1:10.000, Abcam, ab6721), Anti-β-Actin Antibody (1:2000, Santa Cruz, sc-47778) and Goat Anti-mouse IgG-HRP (1:10.000, Santa Cruz, sc-2005). The immunoblot was visualised with chemiluminescence (Clarity™ Western ECL Substrate, 1705060, BioRad).

### 2.12 Statistical tests

Statistical analyses were performed using GraphPad Prism software by one-way ANOVA with Dunnett's post-hoc comparison test where *p* values <0.05 were considered significant. For thermostability shift values (Δ*T*_m_), individual *T*_m_ control values were subtracted from *T*_m_ control averages to give the control Δ*T*_m_ zero value with standard deviation for comparison with test values. For HEK293 cell extracellular flux measurements, statistical significance was determined by two-way ANOVA and Holm-Sidak post-hoc analysis (∗*p* < 0.05).

## 3 Results

### 3.1 UCP1 thermostability shifts detect a specific destabilising interaction with activators

**Figure 1:**
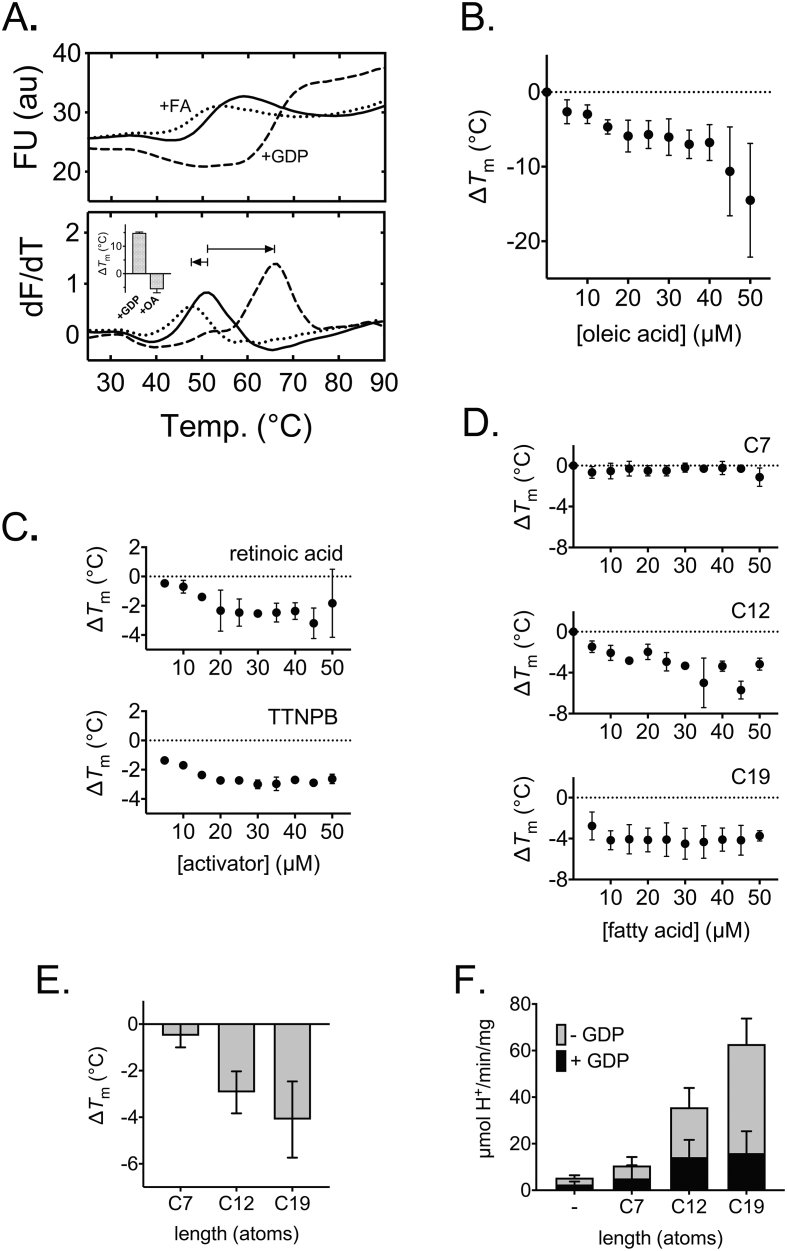
Activators induce a specific destabilisation of native UCP1. The relative thermal stability of purified UCP1 monitored by the fluorescence of CPM-adduct formation at cysteine residues as they become solvent-exposed due to thermal denaturation (see Methods). (A) The thermal denaturation profile (top), and corresponding first derivative (bottom), of native UCP1 in assay buffer with 0.1% 12MNG detergent in the absence (solid line) or presence of 1 mM GDP (dashed line) or 25 μM oleic acid (dotted line). *Inset*, average (±SD) shift in UCP1 ‘melt temperature’ (Δ*T*_m_; condition minus control) associated with either ligand. (B–D) Corresponding shifts in UCP1 thermal stability associated with the addition of the indicated concentration of oleic acid (B), retinoid activators (C) or saturated fatty acids of differing carbon alkyl chain length (D, and E, at 25 μM specifically; C7, heptanoic acid; C12, dodecanoic acid; C19, nonadecanoic acid). (F) The corresponding proton uptake rates (nmol H^+^/min/mg protein) by UCP1 reconstituted into liposomes in the absence or presence of 100 μM C7, C12 or C19, with and without 1 mM GDP, as indicated. Data are averages (±SD) of 3–4 independent experiments for Δ*T*_m_ values and 4 independent experiments for proton uptake rates.

We have applied a rapid fluorescence-based assay to assess the relative thermostability of purified UCP1, which can provide practical information on membrane protein integrity and interaction with detergent, lipid and ligands [29]. Samples in the assay are subjected to a temperature ramp while protein unfolding is monitored by an increase in fluorescence of the probe *N*-[4-(7-diethylamino-4-methyl-3-coumarinyl)phenyl]maleimide (CPM), which reacts with protein thiols as they become solvent exposed due to denaturation to give a fluorescent adduct (cf. [28]). When assessed, UCP1 purified in dodecyl maltose neopentyl glycol (12MNG) detergent exhibited a background fluorescence consistent with at least one solvent-exposed cysteine residue present in the native state, with a transition to a higher plateau as remaining buried cysteines are revealed during protein unfolding (Figure 1A, *top*), as observed previously in similar detergents [19,29]. Our sequencing of the ovine UCP1 gene coding region verified the presence of 8 cysteine residues in the protein (Supplementary Fig. 1; rather than 9 reported in wider sequencing data elsewhere, e.g. [33]). The peak in the derivative of the unfolding transition provides an apparent ‘melt temperature’ (*T*_m_) as a relative measure of thermal stability for a given protein population [29] (e.g. 51.0 °C for UCP1, Figure 1A, *bottom*). This measure relates to the strength and sum of the molecular bonding that occurs within and between associated components, which contribute to the overall protein stability. Accordingly, we have found the apparent *T*_m_ of membrane proteins to exhibit distinct shifts (Δ*T*_m_) in the presence of ligands, consistent with reporting on the bonding changes associated with ligand interaction [19,29,34].

GDP increases the stability of UCP1 (e.g. with a Δ*T*_m_ of +15 °C at 1 mM, Figure 1A, *bottom*), where larger stabilisation is observed in conditions where the inhibitor is known to bind more tightly (cf. [29]). In contrast, when tested, we found that the UCP1 activator oleic acid *destabilised* UCP1 (Δ*T*_m_ of −6 °C at 25 μM, Figure 1A, *bottom*). Fatty acids can act as harsh ionic soap at neutral pH and potentially denature proteins non-specifically. Importantly, however, when the oleic acid concentration was varied, the drop in *T*_m_ did not magnify as a simple function of the concentration, as would be expected of a non-specific effect, but instead plateaued towards a Δ*T*_m_ of −6 °C between 20 and 40 μM oleic acid (Figure 1B), consistent with saturation of a specific interaction event that results in net decreased bonding within the UCP1 population. These concentrations are notably higher than those required to induce uncoupling by UCP1 in studies with isolated mitochondria (e.g. ∼1 μM [10]), which potentially relates to the spread of fatty acids across a larger hydrophobic phase present here provided by the detergent micelle population (0.1% 12MNG). Above ∼40 μM oleic acid, a second less consistent phase was observed with an increased drop in stability with oleic acid concentration, likely representing the saturation of the species in the protein-detergent micelles and the onset of generic protein denaturation.

Further tests with other known activators of UCP1 revealed similar trends, consistently showing a destabilising effect. The UCP1 activators retinoic acid and TTNPB (cf. [35,36]) both destabilised UCP1 with a saturating behaviour when assessed over similar concentrations (towards a maximum Δ*T*_m_ of ∼ −3 °C, Figure 1C), as did the long chain fatty acid nonadecanoic acid (C19; towards a Δ*T*_m_ ∼ −4 °C, Figure 1D). The shorter chain and less potent activator of UCP1 dodecanoic acid (C12) also destabilised UCP1 but with smaller Δ*T*_m_ shifts and without a distinguishable saturation profile, whereas the shortest fatty acid tested, heptanoic acid (C7), showed almost no destabilisation at all (Figure 1D and E). Notably, the size of the shift observed had a strong correlation with fatty acid length and ability to activate UCP1, as verified by proton leak activity assays with UCP1 proteoliposomes (cf. Figure 1F and E). These thermostability measurements reveal that a specific destabilisation of purified UCP1 is fundamental to the interaction with activators, and suggests a net reduction in total bonding occurs in the protein population relating to the activation process.

### 3.2 Activators interact with UCP1 as transport substrates

**Figure 2:**
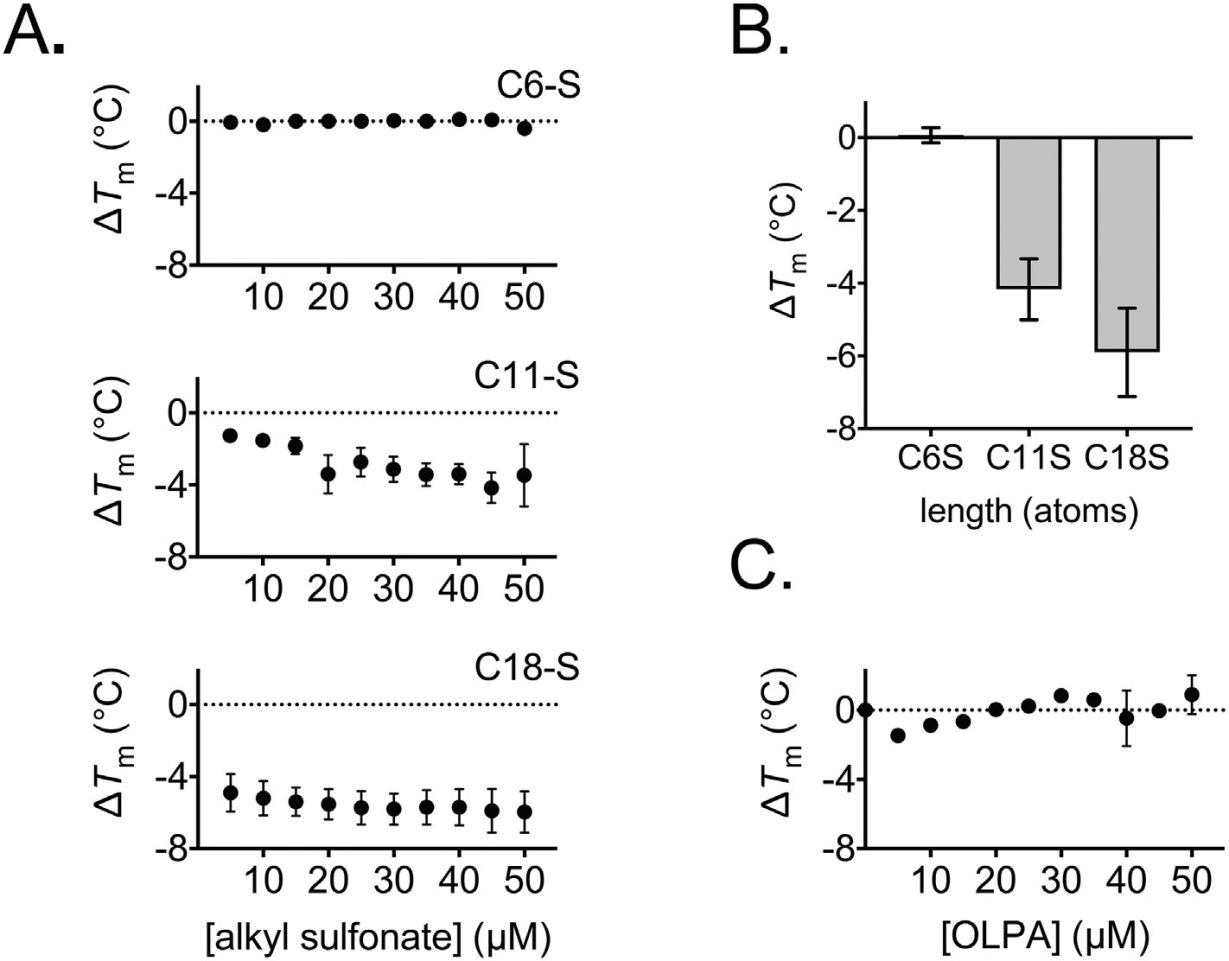
Alkyl sulfonate transport substrates and fatty acids induce the same specific destabilisation of native UCP1. The relative thermal stability of purified UCP1 was determined as described for Figure 1 (see Methods). The shifts in UCP1 thermal stability (Δ*T*_m_) associated with the addition of the indicated concentration of alkyl sulfonates (A) (C6-S, hexane sulfonate; C11-S, undecane sulfonate; C18-S, octadecane sulfonate; equivalent in atom length to C7-C19, cf. Figure 1), or at 25 μM specifically (B), or with the addition of OLPA inhibitor (C), as indicated. Note, C18-S tests included 1.6 mM methyl-β-cyclodextrin solubilising agent. All values given are averages (±SD) of 3 independent experiments.

Ligand–protein binding generates new bonds, hence, an apparent decrease in overall bonding suggests significant conformational changes occur elsewhere in the protein to account for bond loss, such as those that occur in transporter proteins to facilitate substrate translocation (cf. [37]). UCP1 has been proposed to transport fatty acids in some mechanistic models of proton leak (see above), which is supported by the observed transport of the fatty acid analogues alkyl sulfonates by the protein [13,15,38]. In contrast to fatty acids, these species cannot be protonated at physiological pH, having very low pKa values (∼−2), and retain a negative charge that prevents them from diffusing directly across lipid membranes. These anions are transported by UCP1 but, importantly, they do not activate proton leak by the protein [13,15,38]. When tested, we found that they induced almost identical trends in UCP1 thermostability to their equivalent fatty acid counterparts. Similar to C19, octadecane sulfonate (C18-S) destabilised the protein, saturating towards a Δ*T*_m_ of ∼ −6 °C between 20 and 40 μM (Figure 2A). Likewise, the shorter chain variants, undecane sulfonate (C11-S) and hexane sulfonate (C6-S) showed smaller shifts, matching C12 and C7, respectively, where the overall amplitude of the change observed correlated with chain length (Figure 2A and B). In further tests with an alternative long chain fatty acid analogue, oleoyl-l-α-lysophosphatidic acid (OLPA), an inhibitor of UCP1 activity [15], only minor shifts were observed in contrast to C18-S and C19 (Figure. 2C). These correlations indicate that activators destabilise UCP1 by interacting in an identical manner to transport substrates, rather than as activators of proton conductance *per se*, and that fatty acid anion transport is fundamental to the activation process. Of significance, the related ADP/ATP carrier was also found to be destabilised by micromolar concentrations of transport substrate (ADP) in similar assay conditions [29], suggesting a common substrate interaction process in both of these carriers.

### 3.3 Novel activators of proton leak are found among compounds that destabilise UCP1

**Figure 3:**
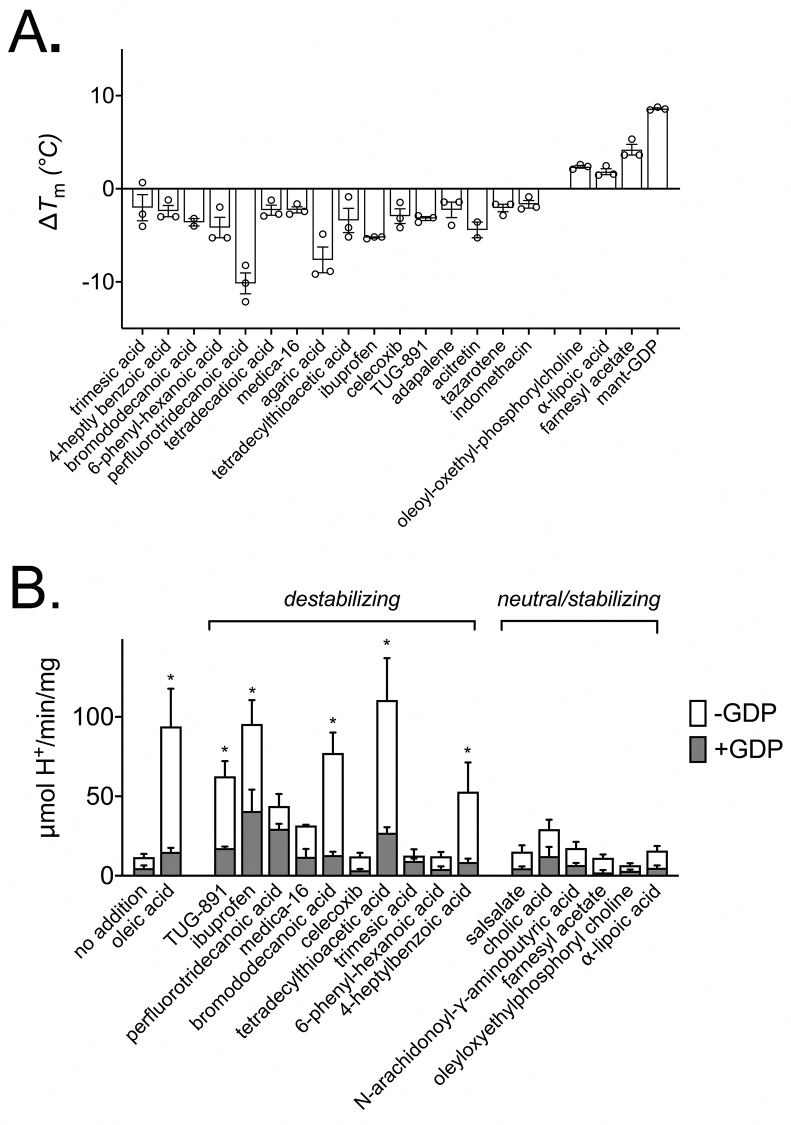
Novel activating ligands are found among compounds that destabilise UCP1 in stability screens. The relative thermal stability of purified UCP1 was determined as described for Figure 1 (see Methods). (A) Interacting ligands that induced a significant shift (Δ*T*_m_) in thermal stability of UCP1 following the screening of 72 notable compounds at 100 μM (see Supp. Fig. 2). Values are averages (±SD) of 3 independent experiments. (B) The proton leak activity (μmol H^+^/min/mg protein) by UCP1 in liposomes (±1 mM GDP) induced by selected compounds following thermostability shift analysis. Activators of proton leak are found specifically among compounds that were identified through a destabilising interaction with UCP1. Values are averages (±SEM) of 3–9 independent experiments. Statistical significance was determined by one-way ANOVA (∗*p* < 0.05).

In light of the distinct stability changes detected with UCP1 activators, we used thermostability shift assays to screen a further 72 compounds, including fatty acid analogues, metabolites and drugs, to identify interacting species of potential significance (Supp. Fig. 2). Compounds were manually selected based on their structural/chemical properties shared with known activators, association in literature with UCP1 or brown adipose tissue, and relevance to mitochondrial metabolism. This approach identified 20 compounds that induced a significant Δ*T*_m_ in UCP1, 16 that were destabilising and four that were stabilising (Figure 3A). Nine of the compounds that destabilised have been reported previously as activating ligands of proton leak or anions transported by UCP1 [15,22,39,40], or were drugs of the retinoid class (adapalene, acitretin and tazarotene) related to the known UCP1 activator retinoic acid [36]. Whereas one of the compounds that stabilised, mant-GDP, a nucleotide derivative, is reported to inhibit UCP1 [23]. The remaining 10 compounds represent potential novel ligands of UCP1.

Compounds of interest were tested in UCP1 proteoliposome assays to determine their impact on proton leak activity. Strikingly, five of the destabilising species tested proved to be activators of UCP1-mediated proton leak, as indicated by significantly increased proton conductance rates relative to controls, which were sensitive to GDP (Figure 3B). In contrast, none of the compounds tested that either increased or had no effect on protein stability activated proton leak activity by UCP1. Nor did any of the non-nucleotide stabilising ligands show significant inhibition of oleic acid-induced proton leak by UCP1 in follow up tests (Supp. Fig. 3). These trends corroborate that the changes in UCP1 responsible for destabilisation are likely an essential feature of activation. Notably, the magnitude of proton leak rates across the various compounds tested did not show a strict correlation with −Δ*T*_m_ shift (Supp. Fig. 4), likely reflecting the occurrence of some interactions related to substrate transport but not proton leak activation specifically (cf. Figure 2). Importantly, the process identified three new compounds as direct activating ligands of the UCP1 protein: tetradecylthioacetic acid, a β-oxidation-resistant synthetic fatty acid that activates peroxisome proliferator-activated receptors (PPAR) and promotes mitochondrial fatty acid oxidation [41]; TUG-891, a G protein coupled receptor 120 (GPR120) agonist reported to stimulate UCP1-dependent mitochondrial uncoupling, fat oxidation and weight loss in mice [42]; and ibuprofen, a widely used non-steroidal anti-inflammatory drug and painkiller [43].

All of the UCP1-activating species observed were hydrophobic (logP >3.5) but amphipathic in nature, with a carboxylate head group that could be protonated at physiological pH to facilitate proton transport (p*K*_a_ >4), consistent with past observations [15,22]. However, the bulk hydrophobic region varied considerably from long chain fatty acids across activators (e.g. TUG-891), suggesting a lack of specific structural constraints. Computational alignment and analysis of verified activators revealed common matching features associated with the carboxylate headgroup for potential polar interactions but no specific features across the bulk region other than general hydrophobicity (see Supp. Fig. 5A). The wider array of UCP1 ligands identified by thermostability analysis alone exhibited similar characteristics but with added diversity in the polar region and no strict requirement for a high-p*K*_a_ carboxylate group (see Supp. Fig. 5B). These findings indicate that UCP1 has a particularly broad ligand specificity, which widens the scope of molecules with potential to activate the protein.

### 3.4 Ibuprofen activates UCP1 activity in isolated brown adipose tissue mitochondria and HEK293 cells but not in immortalised brown adipocytes

**Figure 4:**
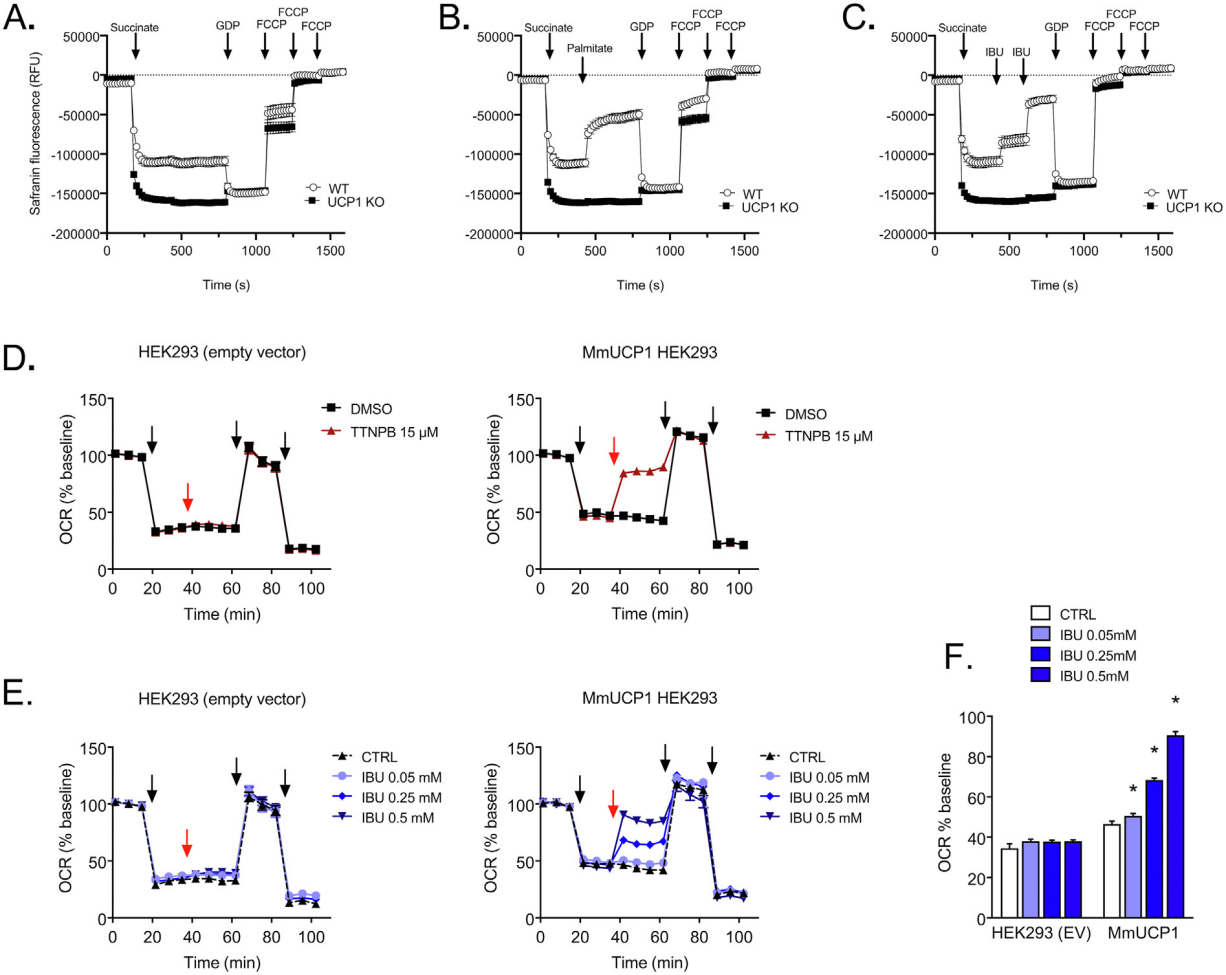
Ibuprofen stimulates UCP1 activity in mitochondria isolated from brown adipose tissue and in MmUCP1 (*Mus musculus*) transfected HEK293 cells. A–C: membrane potential measurements with safranin O for mitochondria isolated from wildtype (WT) or UCP1 KO mice, without (A) or with additions of 100 μM palmitate (B) or 2 × 250 μM ibuprofen (C) and other effectors, as indicated (see Materials and Methods). n = 6 samples per group, measured on two independent days. Results are shown as mean ± SEM. (D and E) Oxygen consumption rate (OCR) of HEK293 cells transfected with an empty vector (EV) or mouse UCP1 (MmUCP1) upon treatment with TTNPB versus DMSO control (D) or different ibuprofen (IBU) concentrations versus buffer control (E). Arrows indicate injections, which are 1. Oligomycin, 2. TTNPB or ibuprofen (red arrow), 3. DNP and 4. rotenone + antimycin A (see Materials and Methods). BSA concentration of medium: 0.4%. (F) Summary of the OCR attributable to proton leak activity (oligomycin-insensitive respiration): treatment with ibuprofen stimulates UCP1 activity in a dose-dependent manner in MmUCP1 transfected HEK293 cells. Values (±SEM) are from 17 to 23 samples per group, measured on four independent days, with statistical significance determined by two-way ANOVA (∗*p* < 0.05). (For interpretation of the references to colour in this figure legend, the reader is referred to the Web version of this article.)

Our screening process revealed that the licenced analgesic ibuprofen can activate isolated UCP1. Further investigations with the more soluble metabolic derivates indicated that only the parent molecule activated the protein in liposomes (Supp. Fig. 6A), in line with the wider observed physicochemical trends. Tests with isolated mitochondria from mouse brown adipose tissue confirmed that ibuprofen could activate UCP1 in the natural membrane environment. Similar to palmitate, the drug decreased the membrane potential of succinate-energised mitochondria from WT mice in a GDP-sensitive manner, but not in mitochondria from UCP1 KO mice (see tests of safranin fluorescence quenching by mitochondria, Figure 4A–C: with no activator, palmitate or ibuprofen addition, respectively). We tested the ability of ibuprofen to activate UCP1 in a cellular environment using both immortalised brown adipocytes from mice and a HEK293 cell UCP1-expression system [35]. Whilst respiratory analysis did not indicate a stimulation of UCP1-dependent respiration in brown adipocytes (Supp. Fig. 6B and C), tests with HEK293 cells transfected with mouse UCP1 (MmUCP1) revealed a dose-dependent stimulation of non-phosphorylating respiration by ≥ 50 μM ibuprofen specific to UCP1-expressing cells (cf. oligomycin-insensitive oxygen consumption rates in UCP1-expressing vs empty vector control cells, Figure 4D–F). The drug was able to induce UCP1-dependent oxygen consumption similar to the TTNPB control, albeit requiring higher concentrations (∼500 μM vs 15 μM TTNPB, cf. Figure 4D–F). These findings provide a proof of principal for our process of identifying novel ligands that have the potential to activate UCP1-dependent energy expenditure in cells. The contrasting lack of response in cultured brown adipocytes suggests that other factors prevent the action of the drug on UCP1 in these cells, such as restricted uptake or a more rapid turnover to inactive variants. Circumventing these factors, e.g. via the development of suitable compound variants, therefore, is a promising new area for further research.

## 4 Discussion

Integral membrane proteins like UCP1 are challenging to study. Hydrophobic ligands, such as fatty acids, can bind non-specifically and potentially act as harsh ionic detergents that denature proteins, hampering binding studies. Furthermore, UCP1 and other mitochondrial carriers are relatively unstable and can be extracted in detergent in an incorrectly folded form, leading to erroneous conclusions on function or biophysical properties (e.g. from common bacterial expression systems; see [29,[44], [45], [46], [47], [48]] and references therein). Here, we have utilised a fluorescence-based protein thermostability assay tailored for membrane proteins to reveal details on the interaction of purified UCP1 with activators. The method reports on stability shifts related to the bonding changes of the protein itself, informing on interacting ligands and potential protein conformational shifts, avoiding issues of conventional binding studies. The methodology is emerging as a useful tool in resolving structural mechanisms and clarifying substrates in other carriers and transport proteins [20,29,34,49,50]. Inherent to the technique is the monitoring of the folded state of the protein, which provides a robust indicator of protein sample integrity. Our experimental approach here not only reveals mechanistic information on how activators influence native UCP1, but also that the protein has a wide ligand specificity and may be directly targeted, independent of fatty acids, for increased energy expenditure.

Our thermostability measurements consistently demonstrated a destabilising interaction of proton leak activators with purified UCP1, which matched the changes observed by equivalent alkyl sulfonate anions that do not facilitate proton leak but are transport substrates of the protein. The common destabilisation profiles suggest the same bonding changes occur and that fatty acids and other activators interact as transport substrates, and that activator transport is fundamental in the UCP1 proton conductance mechanism. As such, our binding study observations support biochemical models of UCP1 proton leak where the protein specifically acts to transport the activating species [13,15] over other models (see [9]). Accordingly, UCP1 exports bound fatty acid anions across the mitochondrial inner membrane, where they return to the matrix in a protonated form, either via UCP1 (*shuttling model* [15]) or by independently flipping directly across the membrane (*cycling model* [13]) to give a net proton conductance. In the former case, long chain fatty acid species are proposed to remain bound to UCP1 during transport in either direction, essentially chaperoning protons. Of the compounds that we observed to destabilise UCP1 that likely represent transport substrates, a subset proved to be activators. Notably, these all had p*K*_a_ values >4, consistent with the notion that it is the inherent ability of the molecule to be protonated in the relevant pH conditions that dictates whether or not a transported species facilitates proton leak, in line with previous evidence in support of these models [15,22].

The distinct loss in stability from activator binding also provides clues to the structural process underlying UCP1 proton leak. The related mitochondrial ATP/ADP carrier exchanges nucleotides by a recently clarified structural mechanism, which is likely conserved across the mitochondrial carrier family of metabolite transporters [20]. The protein cycles between a cytoplasmic (c-state) and matrix (m-state) conformation to alternate access of a substrate binding site within the central cavity of the protein to either side of the membrane for stepwise substrate transport. Substrate-binding induced state shifts occur through symmetrical movements in the three pseudo-symmetrically related domains of the carrier. Notably, deviations from symmetry occur in the central binding site to accommodate the non-symmetrical nature of the substrate. Similar to our observations here with UCP1, the purified ATP/ADP carrier was also *destabilised* by micromolar concentrations of ADP substrate, or by the m-state inhibitor bongkrekic acid, in similar conditions [29], consistent with ligand-induced transitioning to a less stable state of the transport cycle. The similar net bonding loss in UCP1 suggests that activating ‘substrates’ induce similar state shifts, and that activation uses the same core transport mechanism rather than a distinct asymmetrical process, as proposed [21,51], where fatty acid anions translocate on the outside surface of the protein at the lipid–protein interface. UCP1 conserves all of the key structural elements of a conventional carrier mechanism, with no distinct asymmetrical features at the membrane-facing surface to facilitate specific interactions for ligand translocation [9], supporting this rationale. The region in UCP1 corresponding to the substrate binding site of common carriers (see [52]) has a triplet of arginine residues known to influence nucleotide binding [53,54], which have potential to interact with the polar/charged group of activators as well, where hydrophobic residues in the central cavity could accommodate additional hydrophobic interactions. Notably, fatty acid activators and nucleotides have been reported to display competitive behaviour in regulating UCP1 [16].

Our screening approach successfully identified novel activating ligands among compounds that destabilised UCP1. The synthetic GPR120 agonist TUG-891 promotes brown adipose tissue thermogenic activity in mice through GPR120-signalling, but is also reported to induce UCP1-dependent oxygen consumption in isolated mitochondria, suggesting an additional mechanism [42]. Our results corroborate these claims and demonstrate that TUG-891 is a direct activating ligand of the UCP1 protein, despite its structural deviation from conventional fatty acid activators. The positive health attributes observed in mice with this compound (increased fat oxidation, reduces body weight and fat mass) are at least in part likely due to the direct targeting of UCP1. The non-β-oxidisable fatty acid analogue, tetradecylthioacetic acid, which promotes mitochondrial fatty acid oxidation through PPAR activation [41], may also confer health benefits as a hypolipidemic agent via UCP1 targeting given our findings, though this notion requires verification. Surprisingly, we discovered that the cyclooxygenase inhibitor and common painkiller ibuprofen is an activating ligand of UCP1 as well, and could activate the protein in isolated brown fat mitochondria and HEK293 cells but not brown adipocytes. In HEK293 cells, cellular activation of UCP1 required 50–500 μM ibuprofen to achieve an 1.1- to 2.0-fold increase in UCP1-dependent respiration (cf. Figure 4F). These concentrations overlap with the ∼70–240 μM therapeutic range of the drug that occurs in human blood plasma/serum associated with its conventional use (relating to a low apparent volume of distribution of ∼0.1–0.2 L/kg) [55,56]. As such, appreciable stimulation of thermogenesis might be possible if a similar UCP1 activation could be achieved in brown adipose tissue. The factor(s) responsible for the absent response in brown adipocyte cultures are not known, but could be due to a rapid sequestration and/or metabolism of ibuprofen specifically in these cells. The generation of ibuprofen-coenzyme A thioesters and incorporation of the drug into triglycerides, as can occur to some degree in white adipocytes [57], may prevent them reaching and activating UCP1. Ibuprofen turnover could also potentially occur via conventional P450 cytochrome related metabolism [55], to give only inactive variants (cf. Supp Fig. 6A). Though in both cases, the metabolism would have to be rapid. Alternatively, the drug may not be taken up by these cells in contrast to kidney cell lines where uptake has been demonstrated to be mediated by an unknown transporter [56]. Ibuprofen transport across the blood brain barrier also relies on an unknown solute carrier, rather than passive diffusion [58]. Hence, the relevant transporters may be limiting in cultured brown adipocytes. Compound variants circumventing these potential barriers (e.g. alternative molecules compatible with anion transporters present in brown adipocyte, or the use of prodrugs to promote uptake and/or passive diffusion), therefore, are an attractive avenue for further investigation.

There is considerable research into methods to encourage brown fat proliferation and the browning of white adipose tissue, though in the absence of physiological stimuli, UCP1 must still be activated to benefit from the full glucose and triglyceride turnover capacity of the tissue [59]. Pharmacological activation of upstream beta-adrenergic targets has been demonstrated in humans but lacks tissue specificity and is complicated by wider systemic effects [6,60]. As a specific, defining feature of brown fat, UCP1 is an attractive target for the therapeutic activation of thermogenic energy expenditure. Our assessment utilising thermostability shift analysis provides an effective route to probe for UCP1-interacting molecules and reveals that the protein can be directly activated by conventional drug-like compounds with relatively wide structural specificity, which opens up the array of candidate molecules with potential to target it.

## Author contributions

R.C. and P.G.C. designed research and wrote the paper; R.C., M.K.H., C.A.C., S.K. and P.G.C. performed research; M.J., Y.L. and E.R.S.K. contributed materials and expertise; R.C., M.K.H., C.A.C., M.J., S.K. and P.G.C. analysed data; all authors contributed to editing.

## Data availability

The data supporting the findings of this study are presented within the article and its supplementary materials, and are available upon reasonable request.
