# Supplementary material for: Activating ligands of Uncoupling protein 1 identified by rapid membrane protein thermostability shift analysis
Source: Mol Metab. 2022 Jun 9;62:101526. doi: 10.1016/j.molmet.2022.101526 (PMC9243162; doi:10.1016/j.molmet.2022.101526)
Supplement: Multimedia component 1 [file mmc1.docx]

**Supplementary Figures to:**

**Activating ligands of Uncoupling protein 1 identified by rapid membrane protein thermostability shift analysis**

Riccardo Cavalieri, Marlou Klein Hazebroek, Camila A. Cotrim, Yang Lee, Edmund R. S. Kunji, Martin Jastroch, Susanne Keipert and Paul G. Crichton

**Ovis_aries_UCP1**

ATGGTGGGACACGCAGCGACAGACGTGCCCCCTACCATGGCAGTCAAGATCTTCTCGGCGGGGGTGGCGGCCTGCGTGGCTGACATAATCACCTTCCCGCTGGACACCGCCAAAGTCCGGCTGCAGATCCAGGGCGAATGCCTGACCTCCAGTGCCTTTAGG**TAT**AAAGGTGTCCTGGGAACAATCATCACTCTGGCAAAAACAGAAGGGCCCGTGAAACTCTACAGCGGGCTGCCTGCTGGTCTCCAGAGACAAATAAGCTTTGCTTCTCTCAGGATCGGCCTCTATGATACTGTCCAGGAGTTCTTCACCACAGGGAAAGAAGCTAGTTTAGGAAGCAAGATCTCAGCGGGCCTAACGACTGGAGGCGTGGCCGTGTTCATTGGGCAACCCACAGAGGTGGTCAAGGTCAGACTGCAAGCACAGAGCCATCTCCACGGTCCCAAACCTCGATACACTGGGACTTACAATGCTTACAGGATTATAGCAACAACAGAAGGCTTGACGGGGCTTTGGAAAGGGACTACTCCCAATCTGACAAGAAATGTCATCATCAACTGTACAGAGCTCGTAACGTATGACCTAATGAAGGAGGCCCTTGTGAAAAACAAACTATTAGCAGACGACGTGCCCTGCCACTTCGTGTCCGCTGTTGTTGCTGGATTCTGCACAACGGTTCTGTCCTCGCCAGTGGATGTGGTGAAAACCCGATTTGTTAACTCTTCACCAGGACAGTACACAAGTGTGCCCAACTGCGCAATGATGATGCTCACTAGGGAAGGGCCGTCAGCTTTTTTCAAAGGATTTGTACCTTCCTTCTTGCGACTGGGATCCTGGAACATCATCATGTTTGTGTGCTTCGAACAGCTGAAGCGAGAATTGATGAAGTCGAGGCAGGCCATGGACTGTGCGACCTAG

**>Ovis_aries_UCP1**

MVGHAATDVPPTMAVKIFSAGVAACVADIITFPLDTAKVRLQIQGECLTSSAFR**Y**KGVLGTIITLAKTEGPVKLYSGLPAGLQRQISFASLRIGLYDTVQEFFTTGKEASLGSKISAGLTTGGVAVFIGQPTEVVKVRLQAQSHLHGPKPRYTGTYNAYRIIATTEGLTGLWKGTTPNLTRNVIINCTELVTYDLMKEALVKNKLLADDVPCHFVSAVVAGFCTTVLSSPVDVVKTRFVNSSPGQYTSVPNCAMMMLTREGPSAFFKGFVPSFLRLGSWNIIMFVCFEQLKRELMKSRQAMDCAT

**
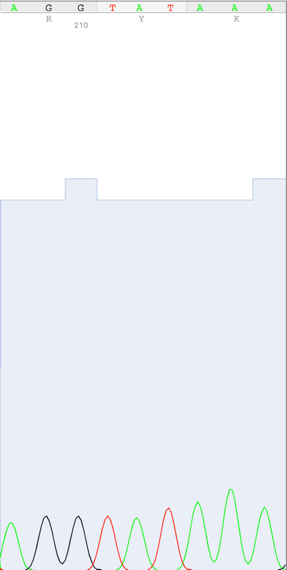
**

**Supplementary Figure 1**. The gene coding sequence (top) and corresponding amino acid sequence (middle) of ovine UCP1 determined by exon sequencing as described in the methods section. Tyrosine 55 (corresponding to the TAT codon), in bold, matches the tyrosine conservation at this position in UCP1 in other species, but contrasts with a cysteine (TGT codon) at this position in other database submissions for the ovine protein (e.g. [[33](#_ENREF_33)]; see [[19](#_ENREF_19)] for example amino acid sequence alignment). The raw sequencing data for the tyrosine 55 codon is also given (bottom)

**

**

**Supplementary Figure 2.** The identification of UCP1 ligands by screening compounds for shifts in protein thermostability. The relative thermal stability of purified UCP1 was determined as described in Methods. 72 compounds were tested at 100 µM to identify interacting ligands that induced a significant shift (Δ*T*_m_; condition minus control) in thermal stability of UCP1. Values shown are averages (±SEM) of 3 independent experiments. Statistical significance was determined by one-way ANOVA tests (**p* < 0.05).

**
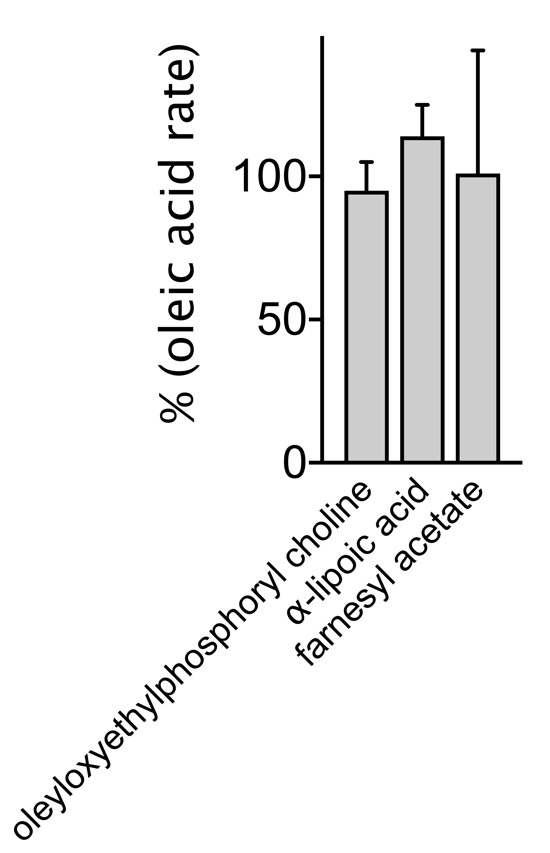
**

**Supplementary Figure 3.** The effect of stabilising compounds on oleic acid-activated rates of proton leak activity. Rates were measured in the presence of 100 µM oleic acid and 100 µM of the indicated compound. Values are expressed as a percentage of the rate obtained in the presence of oleic acid alone and are averages (±SD) of 3-4 independent experiments.


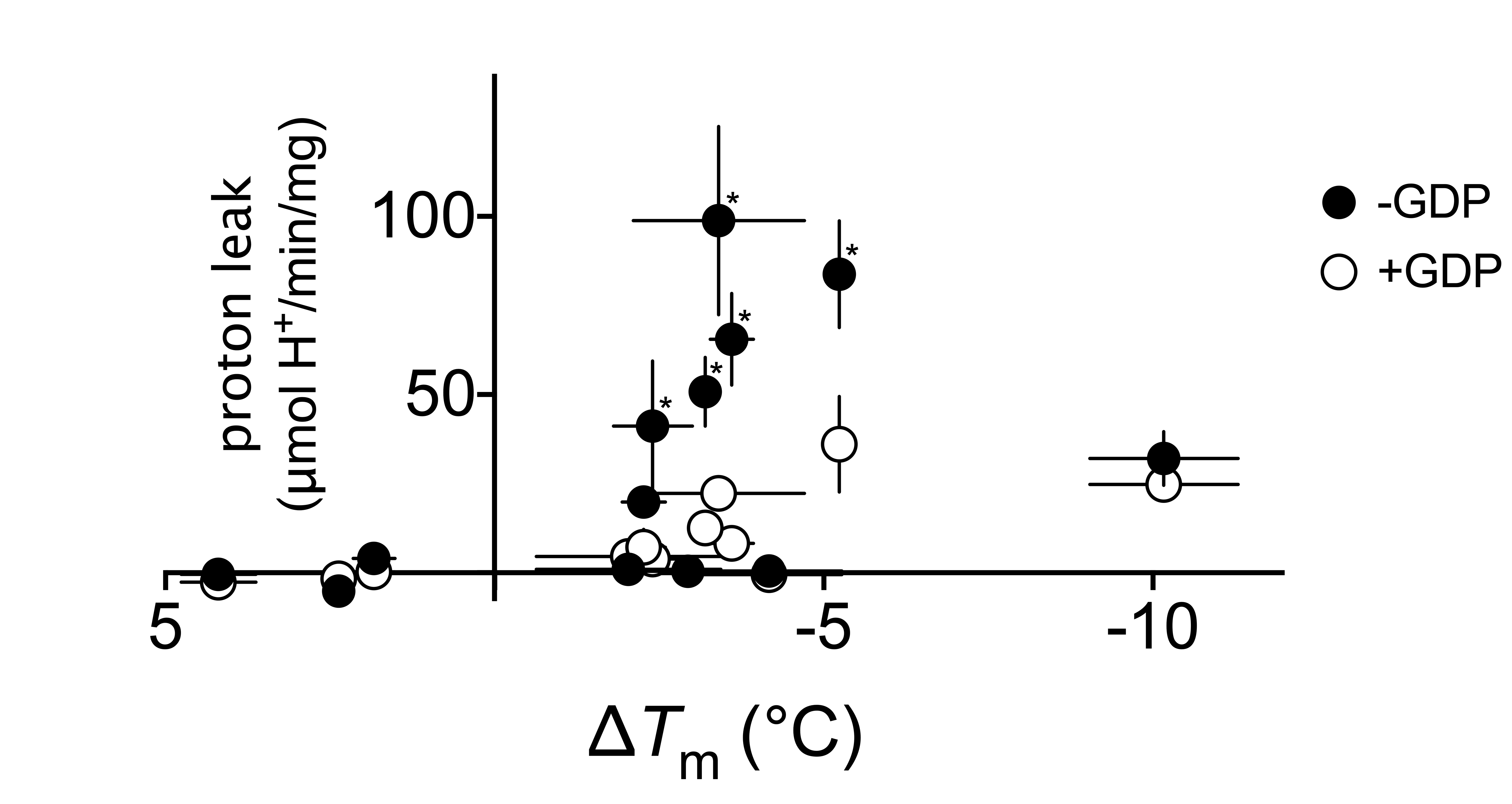


**Supplementary Figure 4**. UCP1 proton leak activation in relation to the corresponding UCP1 destabilisation induced by tested compounds. Rates of proton leak in UCP1 proteoliposomes (±1 mM GDP) in the presence of tested compounds (see Fig. 3B), with background rates in the absence of compound subtracted, and shown as a function of the corresponding UCP1 thermostability shift (Δ*T*_m_) induced by each compound (see Fig 3A). *Compounds that significantly activated UCP1 proton leak (see Fig 3B).


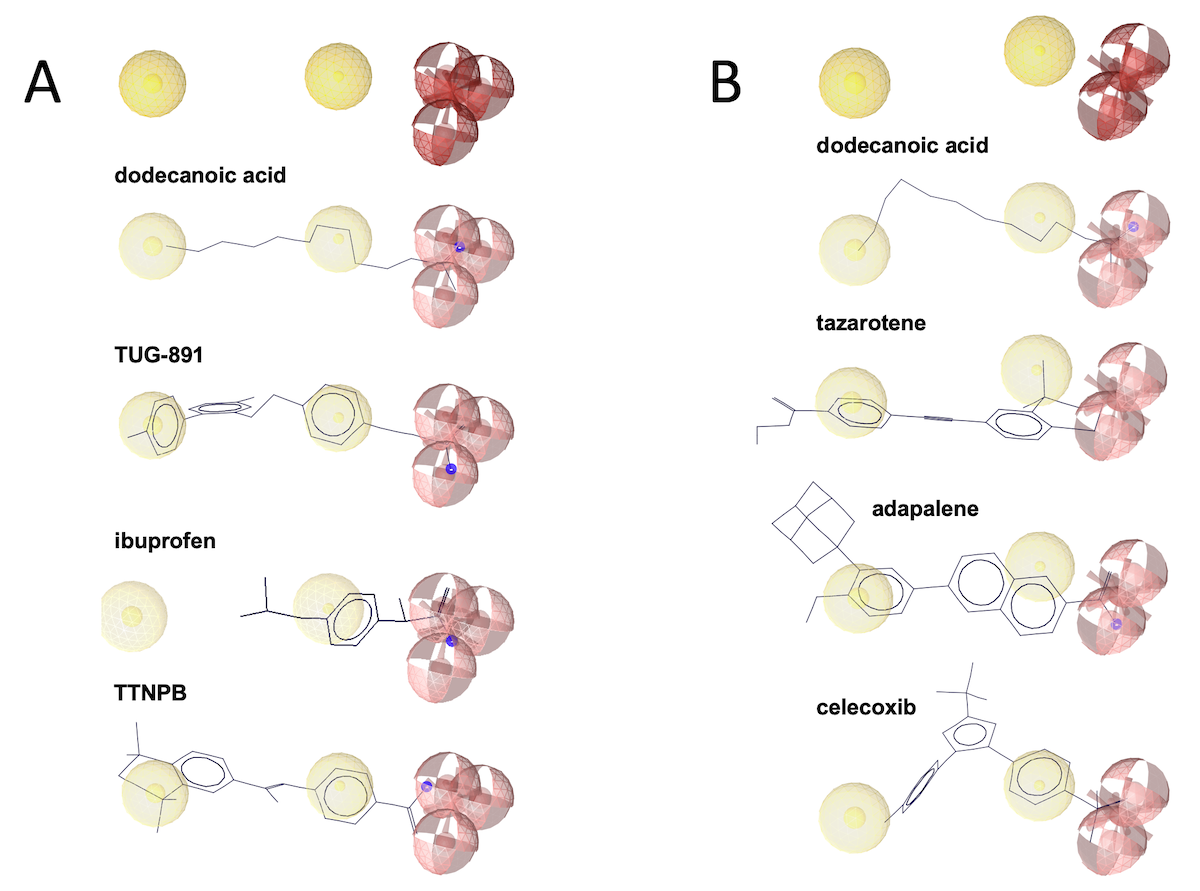


**Supplementary Figure 5.** *In silico* pharmacophore modelling for common features in proton leak activators alone (A) or with the inclusion of wider destabilising ligands (B) of UCP1. Models were generated as described in the Materials and Methods. In each model (top), the following common matching features are shown: hydrogen bond acceptor (red spheres), negative ionizable area (red star) and hydrophobic interactions (yellow spheres). Below each model alignments of example molecules from each group are given (negative charges are shown in blue). Ten activators were used alone or as part of a wider group of 21 destabilising ligands in each case.


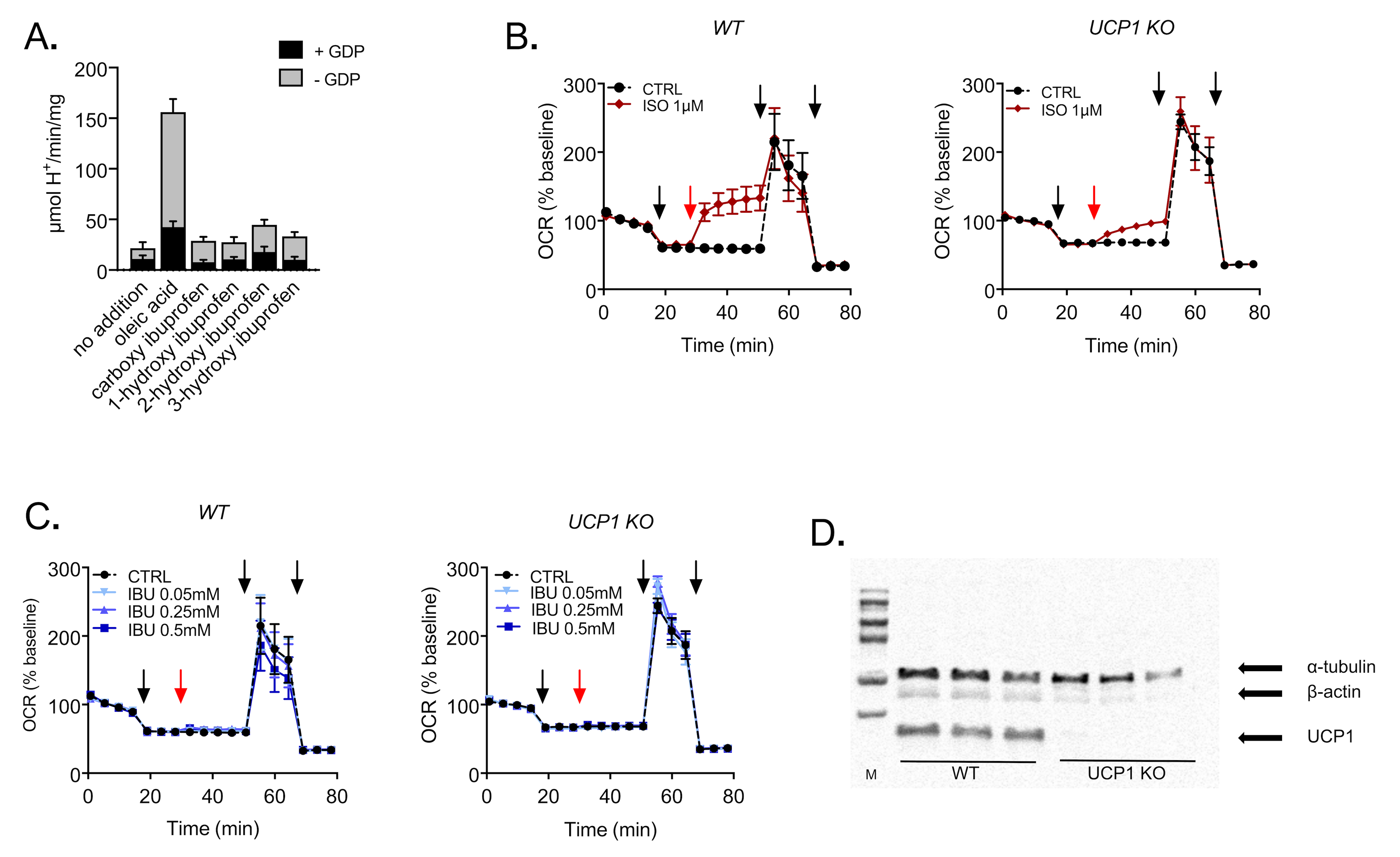


**Supplementary Figure 6.** The effect of ibuprofen derivates on UCP1 activity in liposomes, and ibuprofen on UCP1 activity in immortalised brown adipocyte (iBAT) cell cultures. (A) The metabolic derivates of ibuprofen do not stimulate proton leak activity by ovine UCP1 in liposomes (± 1mM GDP). Values are averages (±SEM) of three to six independent experiments. B and C: ibuprofen does not stimulate UCP1-dependent respiration in iBAT cells. Oxygen consumption rate (OCR) of WT and UCP1 KO iBAT cells upon treatment with isoproterenol versus buffer medium CTRL (B), or ibuprofen versus buffer medium CTRL (C), at the concentrations indicated. n=9 samples per group, measured on three independent days. Arrows indicate injections, which are 1. Oligomycin, 2. Isoproterenol or ibuprofen (red arrow), 3. DNP and 4. Rotenone + Antimycin A (see Materials and Methods). BSA concentration of medium: 0.4%. Values (±SEM) are from 9 samples per group, measured on 3 independent days. D: UCP1 protein expression in WT iBAT cells versus no UCP1 protein expression in UCP1 KO iBAT cells.
